# Lateral-Apical Approach to Pericardiocentesis for Treatment of Cardiac Tamponade Immediately Post-orthotopic Liver Transplantation

**DOI:** 10.7759/cureus.15684

**Published:** 2021-06-16

**Authors:** Christan D Santos, Joshua A Propst, Juan M Canabal, Rohan M Goswami

**Affiliations:** 1 Department of Critical Care Medicine, Mayo Clinic, Jacksonville, USA; 2 Department of Transplant Medicine, Mayo Clinic, Jacksonville, USA

**Keywords:** point-of-care ultrasound, tamponade, liver transplant, orthotopic liver transplant, pericardiocentesis, cardiac tamponade

## Abstract

Cardiac tamponade is a rare complication following orthotopic liver transplantation (OLT). The incidence and treatment specific to the immediate postoperative OLT patient have never been reported. Here, we describe a case of OLT complicated by coagulopathy and difficult intraoperative pulmonary artery catheter placement with subsequent postoperative hemopericardium resulting in tamponade. An emergent, ultrasound-guided, lateral-apical pericardiocentesis was successfully performed, suggesting a possible procedural technique for pericardiocentesis in the immediate postoperative period for liver transplant patients.

## Introduction

Cardiac tamponade following orthotopic liver transplantation (OLT) is an uncommon occurrence that is rarely reported in the literature. Cardiac chamber perforation secondary to pulmonary artery catheter (PAC) insertion is also rare [1-4]. Cardiac tamponade should be considered and confirmed quickly with point-of-care ultrasound (POCUS) in patients with acute cardiogenic shock following either one of these procedures as delay in recognition and management can have dire consequences [5]. Bedside pericardiocentesis was historically performed via the subxiphoid approach. However, ultrasound-guided pericardiocentesis (UGP) has been performed since 1979 and has been widely documented to be safe and efficacious for the management of cardiac tamponade [6]. Here, we present a case of cardiac tamponade post-OLT with successful UGP via a lateral-apical approach.

This article was previously presented as a meeting abstract (abstract number 1702) at the 2020 Society of Critical Care Medicine, 49th Critical Care Congress on February 16, 2020, at the Orange County Convention Center in Orlando, Florida, United States of America.

## Case presentation

A 70-year-old female with renal insufficiency, Crohn’s disease, and alcoholic cirrhosis complicated by ascites, hepatic encephalopathy, and coagulopathy underwent OLT with intraoperative hemodynamic and echocardiographic monitoring via PAC and transesophageal echocardiography. Intraoperative complications included coagulopathy requiring multiple transfusions and difficult PAC placement requiring multiple insertion attempts and readjustments. Postoperatively, the patient was transferred to the intensive care unit (ICU) in stable condition without vasoactive support.

**Figure 1:**
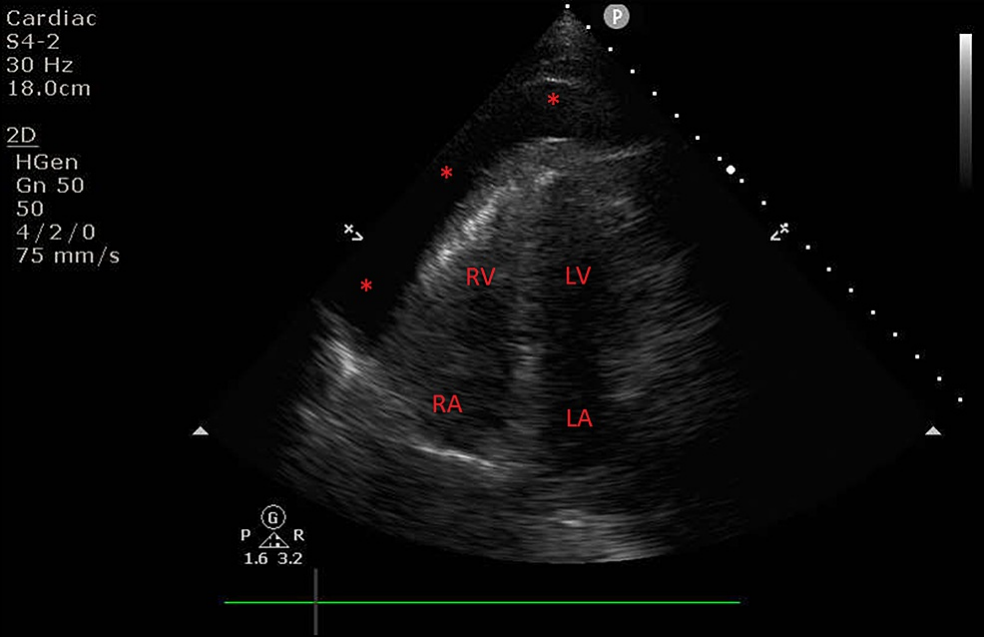
Large pericardial effusion. Transthoracic apical echocardiographic view revealing large pericardial effusion as noted by red asterisks. RA: right atrium; RV: right ventricle; LA: left atrium; LV: left ventricle

Within an hour of arrival, she became tachycardic (110-120 beats per minute) and severely hypotensive (mean arterial pressure of 40-50 mmHg) unresponsive to volume resuscitation. Central venous pressures (CVP) acutely rose to greater than 20 mmHg with near equalization of CVP (20-25 mmHg) and diastolic pulmonary artery pressure (25-30 mmHg). Phenylephrine and epinephrine boluses were attempted without improvement in her hemodynamics. Cardiopulmonary resuscitation was not initiated nor indicated during this period. POCUS revealed a large pericardial effusion with biatrial and biventricular collapse compatible with tamponade physiology (Figure 1).

**Figure 2:**
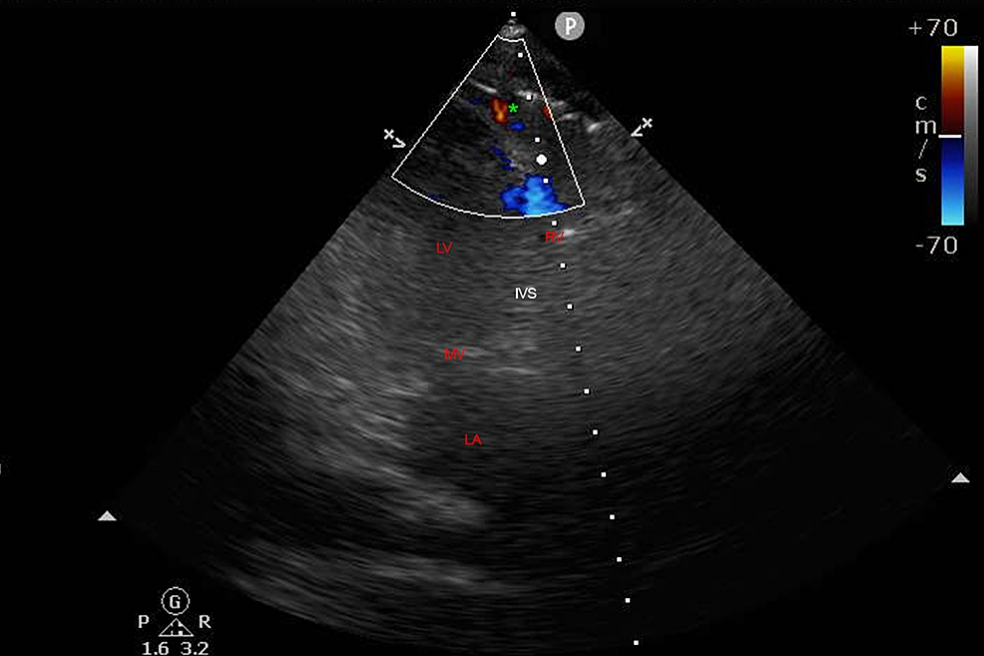
Color Doppler revealing extracardiac flow. Transthoracic echocardiographic view of extracardiac flow as indicated by green asterisk. RV: right ventricle; LA: left atrium; LV: left ventricle; MV: mitral valve; IVS: interventricular septum

Color Doppler revealed a small area of diastolic flow exiting the right atrial (RA) free wall at the RA-inferior vena cava junction concerning for perforation (Figure 2).

Emergent bedside UGP was performed with a lateral-apical approach, as a subxiphoid approach was unavailable due to the size and position of the left lobe of the graft. An 8-French pigtail drainage catheter was placed with immediate return of 350 mL of frank blood and normalization of hemodynamics. Due to acute blood loss, anemia, and deranged thromboelastogram, the patient received two units of red blood cells, 20 units of cryoprecipitate, one unit of platelets, and desmopressin (0.3 mcg/kg). Given the patient’s stability, and after consultation with cardiothoracic surgery, invasive repair was deferred. Serial POCUS revealed stable biventricular function without reaccumulation of pericardial fluid. The drain was removed 48 hours after placement. The patient transferred out of the ICU on postoperative day (POD) three and was discharged home on POD eight. She was followed in the outpatient setting weekly for the month following discharge. She was noted to have normal allograft function, stable renal insufficiency, and no further cardiac complications.

## Discussion

Cardiac tamponade occurs when intrapericardial pressure is elevated due to air or fluid collections which constrain cardiac filling causing life-threatening hemodynamic instability. It is primarily a clinical diagnosis with a constellation of hemodynamic and echocardiographic changes [7]. Cardiac tamponade is rare in OLT patients; however, their inherent thrombocytopenia and coagulopathy increase the risk of hemopericardium in the setting of atrial perforation, as seen in this case.

Invasive hemodynamic monitoring via PAC insertion during liver transplantation varies across facilities. Traditional PACs measured cardiac output by intermittent thermodilution which was limited in accuracy by several user-dependent techniques as well as administration of large volumes of intravenous fluids and cooled blood products during transplantation. The new generation of PACs utilize heat rather than cold thermodilution via a thermal filament connected to the catheter, resulting in continuous cardiac output monitoring [8]. Improvements in algorithms and computational techniques have resulted in the calculation of global end-diastolic volume and the right ventricular end-diastolic and end-systolic volumes, all of which provide a more accurate estimation of intravascular blood volume [9]. Our facility routinely utilizes PACs for hemodynamic monitoring and intravascular volume assessment during liver transplantation. Some facilities reserve this resource only for patients with portopulmonary hypertension, as these patients have been found to have high perioperative mortality [9].

Chamber perforation secondary to PAC insertion is rare, as low as 0.011% according to one clinical audit of 9,071 insertions over 16 years [1-4]. It is difficult to discern if perforation is caused by the guidewire or the catheter itself. However, with routine use of curved tip guidewires, the likelihood of injury caused by the wire is low. In this patient, it is most likely that the perforation occurred during the passage of the PAC from the RA to the right ventricle, perhaps secondary to balloon under-inflation. Delay in symptomology until the postoperative period is likely due to discontinuation of active coagulopathy correction.

Procedural ultrasound guidance has become the standard of care when performing pericardiocentesis as treatment of cardiac tamponade. It has been found to be safe, effective, and easy to perform, with minimal complications and increased success rates, even in patients with coagulopathy and thrombocytopenia [6,10]. Typical approaches include subxiphoid, apical, and parasternal. However, the apical approach is typically avoided due to a higher risk of ventricular puncture [6]. Utilization of POCUS has been found to provide rapid, noninvasive differentiation of shock as well as reduce procedural complications and improve patient safety [11-13].

## Conclusions

In our patient, a lateral-apical approach to UGP was utilized to avoid puncturing the transplanted allograft. The coagulopathic nature of any liver failure patient inherently increases hemorrhagic complications of invasive procedures. With echocardiographic guidance, this patient’s hemopericardium was drained without complication and hemodynamic stability was restored. This case demonstrates patient-specific adaptation of an emergent bedside procedure made possible by ultrasound, further demonstrating the need for POCUS competency among all intensive care providers.
